# Pathotypes and Antimicrobial Susceptibility of *Escherichia Coli* Isolated from Wild Boar (*Sus scrofa*) in Tuscany

**DOI:** 10.3390/ani10040744

**Published:** 2020-04-24

**Authors:** Fabrizio Bertelloni, Giovanni Cilia, Samantha Bogi, Valentina Virginia Ebani, Luca Turini, Roberta Nuvoloni, Domenico Cerri, Filippo Fratini, Barbara Turchi

**Affiliations:** Department of Veterinary Science, University of Pisa, 56126 Pisa, Italy; giovanni.cilia@vet.unipi.it (G.C.); samanthabogi8@gmail.com (S.B.); valentina.virginia.ebani@unipi.it (V.V.E.); luca.turini@phd.unipi.it (L.T.); roberta.nuvoloni@unipi.it (R.N.); domenico.cerri@unipi.it (D.C.); filippo.fratini@unipi.it (F.F.); barbara.turchi@unipi.it (B.T.)

**Keywords:** wild boar, *E. coli*, pathotypes, antimicrobial resistance

## Abstract

**Simple Summary:**

In recent years, in geographic areas such as Central Italy, the wild boar population has greatly increased. Although wildlife represents a fundamental resource for ecosystems and biodiversity, wild animals could act as reservoirs for different pathogens, representing an issue for human and domestic animal health. This investigation reports the presence and circulation of pathogenic and antimicrobial-resistant *Escherichia coli* in wild boar hunted in Tuscany (Italy). Different pathotypes, responsible for human and animal diseases, were detected. Furthermore, resistance to commonly used antibiotics was detected in a large percentage of isolates. Considering the possibility of contact between wild boars, domestic animals, and humans, active monitoring of pathogens is essential for epidemiological purposes.

**Abstract:**

Wild boar are among the most widespread wild mammals in Europe. Although this species can act as a reservoir for different pathogens, data about its role as a carrier of pathogenic and antimicrobial-resistant *Escherichia coli* are still scarce. The aim of this study was to evaluate the occurrence of antimicrobial-resistant and pathogenic *Escherichia coli* in wild boar in the Tuscany region of Italy. During the hunting season of 2018–2019, *E. coli* was isolated from 175 of 200 animals and subjected to antimicrobial resistance tests and PCR for detection of resistance and virulence factor genes. The highest resistance rates were against cephalothin (94.3%), amoxicillin–clavulanic acid (87.4%), ampicillin (68.6%), and tetracycline (44.6%). The most detected resistance genes were *bla_CMY_*_-2_ (54.3%), *sul1* (38.9%), *sul2* (30.9%), and *tetG* (24.6%). Concerning genes encoding virulence factors, 55 of 175 isolates (31.4%) were negative for all tested genes. The most detected genes were *hlyA* (47.4%), *astA* (29.1%), *stx2* (24.6%), *eaeA* (17.1%), and *stx1* (11.4%). *E. coli* was classified as Shiga toxin-producing *E. coli* (STEC) (21.7%), enterohemorrhagic *E. coli* (EHEC) (6.3%), enteroaggregative *E. coli* (EAEC) (5.1%), and atypical enteropathogenic *E. coli* (aEPEC) (3.4%). Enterotoxigenic *E. coli* (ETEC), enteroinvasive *E. coli* (EIEC), and typical enteropathogenic *E. coli* (tEPEC) were not detected. Our results show that wild boars could carry pathogenic and antimicrobial-resistant *E. coli*, representing a possible reservoir of domestic animal and human pathogens.

## 1. Introduction

Wild boar (*Sus scrofa*) is one of the most common wild mammals in the world. In Europe, it is widespread almost throughout the entire continent, and in recent years its population has constantly increased [1]. There are many occasions of interactions between wild boars and humans. Hunting activities, especially in areas where many wild boar are killed every year, such as Central Italy, represent one of the most common causes of interactions not only with humans, but also with hunting dogs [2]. At the same time, in recent years, these animals have been more often spotted in urban and peri-urban zones [3]. Likewise, wild boar frequently come into contact with domestic animals, especially where extensive or semi-extensive breeding is adopted [4,5,6,7].

*Escherichia coli* is a commensal bacterium living in the intestines of animals and humans, but some strains have acquired specific virulence characteristics that allow them to cause several diseases [8,9]. Pathogenic *E. coli* is currently classified into eight pathotypes responsible for gastrointestinal infections. Based on the presence of virulence genes and pathogenic mechanisms, they are distinguished as enteropathogenic *E. coli* (EPEC), Shiga toxin-producing *E. coli* (STEC) or verocytotoxin-producing *E. coli* (VTEC), enterohemorrhagic *E. coli* (EHEC), enteroinvasive *E. coli* (EIEC), enteroaggregative *E. coli* (EAEC or EAggEC), enterotoxigenic *E. coli* (ETEC), diffusely adherent *E. coli* (DAEC), and adherent invasive *E. coli* (AIEC) [9,10,11,12,13]. Commensal and pathogenic strains may also represent a serious hazard with regard to their antimicrobial resistance. Indeed, resistance to the main antibiotic classes among *E. coli* strains frequently occurs worldwide, with different strains even exhibiting multidrug resistance. Circulation of these bacteria among animals could cause infections that are difficult to treat, but could also represent an important risk for human health [14,15]. Recent data indicate that in Europe, commensal *E. coli* among domestic animals resulted in resistance, especially to tetracycline, sulfamethoxazole, ampicillin, and trimethoprim. Furthermore, multidrug resistance to three or more antimicrobial classes was recorded in 34.9% and 27.7% of isolates from pig and calves, respectively [16].

Domestic and wild animals could play important roles as reservoirs for some of these serotypes, in particular EPEC, STEC, EHEC, ETEC, and EAEC [10,11]. Despite the great importance of *E. coli*-related diseases in both human and animal health, epidemiological information on the pathotype distribution in wild boar is still scarce; furthermore, most of the studies are mainly focused on STEC and EHEC. In Europe, many studies were conducted in the Iberian Peninsula, where in Spain, three investigations reported a prevalence of STEC in wild boar of 8.0%, 8.4%, and 3.3% [17,18,19]. Furthermore, one of these studies described the presence of EPEC isolates in three subjects [19]. More recently, a study conducted in Portugal showed 4.8% positivity for STEC in wild boar [20]. In Poland, 28.2% and 30.9% STEC and EPEC, respectively, were isolated from rectal swabs of wild boar [21]. In Italy, a recent investigation carried out on different wild animals identified one EPEC and one EHEC isolate from a small number of wild boar [22]. On the other hand, no positive samples among wild boar were detected for EHEC in a survey carried out in Sweden [23].

A more conspicuous number of studies focused on the antimicrobial resistance of *E. coli* isolated from wild boar. Indeed, in recent years, interest in the role of this particular wild animal in the maintenance and diffusion of antimicrobial-resistant bacteria increased [24]. Two surveys carried out in Spain revealed low levels of resistance (7.5% and 12.7%), especially to sulfamethoxazole, tetracycline, and ampicillin [25,26]. A study conducted in Portugal showed a higher prevalence of resistant *E. coli* from wild boar. In particular, 25% of isolates showed resistance to ampicillin, tetracycline, co-trimoxazole, and streptomycin [27]. Moreover, other research conducted in the same country revealed 10.4% positivity of wild boar to extended-spectrum β-lactamase (ESBL) *E. coli*, associated with resistance to other antimicrobials [28]. In eastern Europe (Serbia, Poland, Czech Republic), recent works revealed the circulation of resistant, multidrug-resistant, ESBL, and cephalosporin-resistant *E. coli* among wild boar, even if in small ratios [29,30,31]. Several studies in Europe reported data about antimicrobial resistance in *E. coli* from wild boar. In particular, carbapenemase-producing *E. coli* were described in Algeria, showing resistance to amoxicillin, amoxicillin–clavulanate, tobramycin, ertapenem, and meropenem [32]. In Brazil, low antimicrobial resistance prevalence was detected, especially to amoxicillin–clavulanic acid (10%), cephalothin (7%), and enrofloxacin (5%) [33]. On the other hand, an investigation carried out in Australia showed moderate resistance to sulfadimethoxine (50.4%) and florfenicol (27.0%), but a low circulation of multidrug-resistant strains (1.7%) [34]. In Italy, information about the antimicrobial resistance of wild boar *E. coli* is limited. Resistance to ampicillin, amoxicillin–clavulanic acid, and streptomycin was recorded in a survey conducted in Central Italy, but only a limited number of samples from wild boar were analyzed [22]. Other authors examined higher numbers of animals, but their work focused only on ESBL, revealing the presence of ESBL-producing *E. coli* in 0.9% of samples [35].

Although in Italy wild boar is the most widespread wild animal, little information is available about the occurrence of pathogenic and antimicrobial-resistant *E. coli*. The aim of this study was to characterize *E. coli* isolates from hunted wild boar in Central Italy in order to identify their pathotypes and antimicrobial resistance profiles.

## 2. Materials and Methods 

### 2.1. Sampling and E. coli Isolation

During the hunting season of 2018–2019 (from November to January), 200 wild boar rectal swabs were collected from the provinces of Pisa, Livorno, Siena, and Grosseto, located in Tuscany, central Italy. All animals included in the study were hunted during the authorized hunting season, following regional hunting law (Regolamento di attuazione della legge regionale 12 gennaio 1994, N. 3 D.P.G.R. 48/R/2017). Sampling was performed just before slaughtering procedures, and within 4 h after collection, swabs were transported to the infectious disease laboratory of the Department of Veterinary Science, University of Pisa. During sampling, the hunting area, sex, and age of each animal were recorded. In particular, age was evaluated after assessing the degree of tooth eruption and wear of teeth of the lower jaw [36]. Swabs were used to inoculate Buffered Peptone Water (Oxoid, Milan, Italy) at 37 °C for 24 h. A loopful from each broth culture was then streaked on Triptone Bile X-glucoronide (TBX) Agar (Oxoid) and incubated at 42 °C for 24 h. From each plate, one single, isolated blue colony was selected, subcultured on tryptone soy agar (TSA) and checked for purity. Pure cultures were grown in Brain Heart Infusion (BHI) broth (Oxoid) at 37 °C for 24 h and, after glycerol addition, frozen at −80 °C.

### 2.2. Detection of Virulence Genes and Determination of Pathotypes 

DNA was extracted from fresh bacterial cultures by using the commercial Tissue Genomic DNA Extraction Kit (Fisher Molecular Biology, Trevose, PA, USA), following the manufacturer’s guidelines. Two multiplex PCRs were performed, using primers (Supplementary Materials Table S1) and protocols previously described for detection of the following genes: *escV, ent, eaeA, bfpB, stx1, stx2, hlyA, saa, invE, astA, aggR, pic, elt, estIa*, and *estIb* [37,38]. Pathotypes were determined in accordance with previously reported guidelines [38].

### 2.3. Evaluation of Antimicrobial Resistance

In accordance with European Committee on Antimicrobial Susceptibility Testing (EUCAST) recommendations (EUCAST disk diffusion method for anti-microbial susceptibility testing version 6.0), the disc diffusion method was employed to determine resistance to the following antimicrobials: ampicillin (AM, 10 mg), amoxicillin–clavulanic acid (AMC, 20–10 mg), cefoxitin (FOX, 30 mg), cephalothin (KF, 30 mg), cefotaxime (CTX, 30 mg), chloramphenicol (C, 30 mg), tetracycline (TE, 30 mg), trimethoprim–sulfamethoxazole (SXT, 19:1, 25 mg), enrofloxacin (ENR, 10 mg), gentamicin (CN, 10 mg), streptomycin (S, 10 mg), imipenem (IPM, 10 mg), and aztreonam (ATM, 30 mg) (Kairosafe Srl, Trieste, Italy). Antimicrobial resistance test was performed on Mueller–Hinton agar plates incubated at 35 °C for 16–20 h and interpreted according to breakpoints provided by EUCAST or CLSI (The Clinical and Laboratory Standards Institute) [39,40].

### 2.4. Detection of Antimicrobial Resistance Genes

The presence of *bla*_CMY2_ (resistance to β-lactam antibiotics), *aadA1* and *strA-strB* (resistance to streptomycin), *tetA*, *tetB*, and *tetG* (resistance to tetracycline), and *sul1*, *sul2*, and *sul3* (resistance to sulfonamide) genes was evaluated by single endpoint PCRs using primers and protocols previously described (Supplementary Materials Table S2) [41,42,43,44,45].

## 3. Results

### 3.1. E. coli Isolation

A total of 175 *E. coli* pure cultures were obtained from the 200 tested animals; 76 from males and 99 from females. Regarding age, 68, 26 and 81 isolates were from young, sub-adult and adult wild boar, respectively. In all, 67 positive samples were collected in Grosseto, 53 in Pisa, 45 in Siena and 10 in Livorno.

### 3.2. Detection of Virulence Genes and Determination of Pathotypes 

A total of 69.1% isolates presented at least one virulence gene. The most detected genes were *hylA* (47.4%), *astA* (29.1%), *sxt2* (24.6%), *eaeA* (17.1%), and *stx1* (11.4%). A low percentage of *E. coli* cultures were positive to *pic* (6.9%), *escV* (4.6%) *aggR* (3.4%), and *saa* (1.7%) genes. None of the tested isolates carried the genes *ent, bfpB, invE, elt, estIa,* or *estIb* (Table 1). No statistical differences were recorded in relation to sex, age and province of sampling, except for *pic*, which was mostly detected in isolates from Siena and *stx1*, mainly present in *E. coli* from sub-adult wild boar.

Based on virulence gene detection, each isolate was classified as a specific pathotype. Specifically, 38/175 isolates were EHEC, 13/175 were STEC, 10/175 were EAEC, and 8/175 were atypical enteropathogenic *E. coli* (aEPEC). Moreover, 16/175 isolates showed an unspecific profile common to two or three pathotypes (Table 2). Lastly, 38/175 *E. coli* could not be classified, because they showed only *hylA* or *astA* virulence genes. No statistical differences were observed in the distribution of pathotypes in relation to sex, age and sampling area.

Shiga toxin-producing *E. coli* (STEC) (21.7%), enterohemorrhagic *E. coli* (EHEC) (6.3%), enteroaggregative *E. coli* (EAEC) (5.1%) and atypical enteropathogenic *E. coli* (aEPEC) (3.4%) were found. Enterotoxigenic *E. coli* (ETEC), enteroinvasive *E. coli* (EIEC) and typical enteropathogenic *E. coli* (tEPEC) were not found.

### 3.3. Evaluation of Antimicrobial Resistance

High levels of antimicrobial resistance were detected in *E. coli*, in particular for β-lactam compounds (Table 3). Indeed, 165/175 isolates were resistant to cephalothin, 152/175 to amoxicillin–clavulanic acid and 120/175 to ampicillin. Many isolates were also resistant to tetracycline (78/175). On the other hand, lower antimicrobial resistance was detected for enrofloxacin and gentamicin (24/175), while low resistance was detected for trimethoprim–sulfamethoxazole (3/175) and chloramphenicol (1/175). None of the isolates showed resistance to imipenem. No statistical differences were observed in relation to sex, age, and province, except for resistance to amoxicillin–clavulanic acid, while *E. coli* isolated from adult wild boar more frequently showed resistance to this antibiotic.

### 3.4. Detection of Antimicrobial Resistance Genes

The gene *bla_CMY_*_-2_ was detected in 95/175 isolates. With regard to tetracycline resistance genes, *tetA* and *tetB* were each found in 29/175 isolates, whereas 43/175 of isolates were positive for *tetG*. In addition, 7/175 *E. coli* showed both *tetA* and *tetG*, 8/175 *tetB* and *tetG,* and 1/175 *tetA* and *tetB*. Finally, 3/175 isolates harbored all tetracycline resistance genes. Many isolates carried *sul* genes; in particular, 68/175 showed *sul1*, 54/175 *sul2*, and 4/175 *sul3*. Regarding sulfonamide resistance gene combinations, 22/175 *E. coli* harbored *sul1* and *sul2*, 2/175 *sul2* and *sul3*, and 1/175 all three genes. The gene *aadA1* was detected in 19/175 isolates and *strA-strB* in 10/175 isolates. None of the isolates were positive for both of these genes.

## 4. Discussion

In this investigation, the most detected pathotypes were STEC (21.7%) and EHEC (6.3%). Enterohemorrhagic *E. coli* represents a subgroup of Shiga toxin-producing *E. coli* harboring the virulence gene *eae*, commonly associated with enteropathogenic *E. coli*, responsible for the typical attaching and effacing lesions. These pathogenic *E. coli* play an important role in human health; indeed, in Europe, STEC infections represent the third highest incidence of zoonosis, with more than 8000 human cases reported in 2018 [46]. STEC outbreaks are generally associated with the consumption of milk or bovine meat [47], as cows are considered the principal reservoir of this bacterium [48]. However, some studies showed circulation of this pathotype in wild animals too [10]. In particular, concerning wild boar, our results are not dissimilar from those of other studies carried out in Europe, where the percentage of positive animals ranged between 3% and 30% [17,18,19,20,21,22]. In the present study, the most detected gene coding for Shiga-like toxins was *stx2*. This is in line with other investigations conducted in Europe on wild boar [17] and in Italy on wild mammals sharing the same ecosystem [22]. However, it is interesting to note that a study carried out on birds in the same geographic area reported *stx1* as the most common gene [49], suggesting a possible clustering of strains in relation to animal species. Of the isolates, 5.7% and 4.6% were EAEC and EPEC, respectively. In both cases, isolates could be classified as “atypical” because they did not present either *aggR* or *bfpB* genes. Typical EPEC (tEPEC) are well documented human pathogens, whereas the role of atypical EPEC (aEPEC) in human health remains unclear [50], even if some clinical cases were recently recorded, especially in children [51]. Animals are considered as potential reservoirs for aEPEC [52,53]. Indeed, our results identified wild boar as a possible carrier, with 4.6% positive animals. Regarding EAEC, only typical EAEC harboring *aggR* genes are considered human pathogens. Generally, animals are not involved in the epidemiology of those strains, they are only carriers of atypical EAEC [54]. This study confirms this hypothesis and shows the circulation of this pathotype among a feral pig population. Interestingly, the *aggR* gene was detected in six *E. coli* isolates, but they were not classified as EAEC because they also carried virulence genes common to other pathotypes. Approximately 9% of the isolates harbored genes associated with more than one pathotype, and for this reason, it was not possible to attribute them to a definite category (Table 2). The classification of *E. coli*, initially linked to clinical or anatomopathological aspects, was simplified and not strict. However, it is likely that a single strain can harbor virulence genes of two different pathotypes. A typical example is EHEC, which can carry virulence genes of EPEC and STEC [8]. Interestingly, in 2011 in Central Europe, a large and severe outbreak of *E. coli* occurred caused by a strain with genes of both EAEC and STEC [55]. Detection of adherent and invasive *Escherichia coli* (AIEC) was not a primary goal of this investigation; however, a recent study by Camprubí-Font and colleagues suggested a screening method to distinguish AIEC isolates [56]. Carrying the *pic* gene and being resistant to ampicillin could represent characteristic traits enabling the identification of this pathotype with an accuracy of 75.5%. In this investigation, a total of six *E. coli* isolates presented these features (Table S3) and they can probably be classified as a potential AIEC. Although more specific tests are needed to confirm our hypothesis, this finding could help to clarify some epidemiological aspects related to this recently classified pathotype, which could be involved in the complex etiology of Crohn’s disease (CD) [12]. None of the isolates belonged to ETEC or EIEC; which is in line with other investigations. Indeed, these pathotypes are not frequently isolated from asymptomatic animals [10,22,57].

A high level of antimicrobial resistance was recorded for β-lactams and tetracycline. Regarding the former, resistance was found for cephalothin (94.3%), amoxicillin–clavulanic acid (86.9%), and ampicillin (68.6%). Even if these percentages seem uncommon and worrying, in Italy, different authors detected similar levels of resistance among Enterobacteriaceae from wild animals other than wild boar [22,58,59,60,61,62]. Although only a few large-scale studies on wild boar *E. coli* antimicrobial resistance are so far available, resistance to β-lactams is commonly detected [25,26,27,28]. Almost half of our isolates (44.6%) showed resistance to tetracycline. Resistance to this antimicrobial has been frequently found in pathogens and commensal bacteria from domestic [16,63,64,65,66] and wild [58,60,67] animals. Even if β-lactams and tetracycline belong to different antibiotic classes, there could be some shared considerations. Since both antibiotics are commonly and largely used in humans and in veterinary medicine, high resistance can generally be found in bacteria isolated from humans and animals [16]. Evidence of their extensive and sometimes improper use was documented by detection of these antimicrobials and resistant bacteria in river water, solid waste, and urban gardens [68,69,70]. Furthermore, a recent review of antimicrobial resistance in wildlife highlighted that generally, omnivores carry antimicrobial-resistant bacteria more often than carnivores and herbivores [71]. Wild boar is an omnivorous animal that shares habitats with domestic animals and has recently become a synanthropic or semi-synanthropic species. For these reasons, they frequently come in contact with humans and “accumulate” antimicrobials and/or antimicrobial-resistant bacteria. On the other hand, a low level of resistance was detected for some other antimicrobials, especially quinolones, aminoglycosides, phenicols, and recently introduced β-lactams. These results showed a limited circulation of resistance to these antimicrobials among wild boar, and consequently in the investigated ecosystem.

Our results indicate that the most detected gene was *bla_CMY_*_-2_ (54.3%), which confers resistance to penicillins, extended-spectrum cephalosporins, and aztreonam, but not to carbapenems. The encoded enzymes are poorly inhibited by β-lactamase inhibitors [72]. The frequent detection of this gene could partly explain the high resistance to β-lactams, including associated amoxicillin–clavulanic acid; all but two *bla_CMY_*_-2_ positive *E. coli* were resistant to one or more of these antibiotics. However, since 45.7% of phenotypically resistant isolates did not harbor *bla_CMY_*_-2_, resistance was probably related to other, not investigated mechanisms. As expected, all *bla_CMY_*_-2_ positive isolates were susceptible to imipenem. 

As regards tetracycline-resistance genes, *tetG* was the most commonly detected (24.6% positive samples), although *tetA* end *tetB* are generally more frequently observed in *E. coli* [73,74,75]. A recent investigation conducted on wild mammals ranging in the same area (fox, wild goat, and wolf) reported that *tetA* and *tetB* were more common than *tetG* [22]. Our results suggest the circulation of different strains and/or resistance genes among different animal species living in the same ecosystem. Interestingly, not all resistant isolates possessed the targeted genes, suggesting the possible circulation of different tetracycline-resistance genes. On the other hand, some *tet*-positive *E. coli* did not show phenotypic resistance, highlighting how some isolates cannot express the resistance gene. These conflicting results stress the importance of both phenotypic and genotypic approaches in antimicrobial resistance evaluation.

Sulfonamide resistance genes were widely detected in tested isolates (57.1% of tested *E. coli* scored positive for at least one gene), such as *sul1* (38.9%) and *sul2* (30.9%). These antibiotics are frequently used, especially in veterinary medicine, and consequently this result was expected.

Few strains scored positive for aminoglycoside resistance genes, whose presence is not commonly associated with phenotypic resistance. These data confirm a low level of resistance against aminoglycosides in *E. coli* from wild boar.

## 5. Conclusions

Wild boar is one of the most important wild animals in Central Italy; indeed, in this geographic area, it reaches a high population density and is one of the most hunted animals, with tens of thousands of animals killed every year. Most surveys have focused on the presence of important zoonotic bacterial or viral diseases in wild boar. However, in Italy, as in Europe, studies concerning the diffusion of *E. coli* pathotypes in this species are still limited. This is also the case for antimicrobial resistance: few works are currently available, and they generally focus on a small number of specimens or a specific antimicrobial class [22,25,26,27,28,29,30,31,35]. This research highlights that wild boar can act as carriers for different *E. coli* pathotypes and antimicrobial-resistant strains. This species shares habitats with other wild animals; nevertheless, it could come in contact, directly or indirectly, with domestic animals, especially in areas where extensive or semi-extensive breeding is adopted. Furthermore, in recent years, the scavenging behavior of wild boar pushed them into peri-urban and urban areas, sometimes in cross-contact with humans. Finally, in Italy, hunted animals are frequently used for people’s own consumption, and consequently, post mortem examinations by official veterinary inspectors are not carried out. In addition, evisceration is often done directly by hunters and does not comply with basic hygienic procedures. In this context, the possibility of directly or indirectly spreading pathogenic and antimicrobial-resistant *E. coli* to humans or animals cannot be ignored. 

## Figures and Tables

**Table 1:** Number and percentage of isolates positive to virulence genes.

Gene	Positive Isolates	
	No.	%
*hylA*	83	47.4
*astA*	51	29.1
*stx2*	43	24.6
*eaeA*	30	17.1
*stx1*	20	11.4
*pic*	12	6.9
*escV*	8	4.6
*aggR*	6	3.4
*saa*	3	1.7
*ent*	0	0.00
*bfpB*	0	0.00
*invE*	0	0.00
*elt*	0	0.00
*estIa*	0	0.00
*estIb*	0	0.00

**Table 2:** Pathotypes detected among *E. coli* isolates from wild boar. STEC, Shiga toxin-producing *E. coli*; EHEC, enterohemorrhagic *E. coli*; EAEC, enteroaggregative *E. coli*; aEPEC, atypical enteropathogenic *E. coli*.

Pathotypes	Isolates	
	No.	%
No virulence genes	54	30.9
Not classifiable	38	21.7
STEC	36	21.7
EHEC	13	6.3
EAEC	10	5.7
aEPEC	8	4.6
EAEC/aEPEC	10	5.7
STEC/EAEC	3	1.7
EHEC/EAEC	2	1.1
EHEC/EAEC/aEPEC	1	0.6

**Table 3:** Antimicrobial resistance in *E. coli* isolates from wild boar.

Antibiotic	Isolates					
	Susceptible		Intermediate		Resistant	
	No.	%	No.	%	No.	%
AM	55	31.4	0	0.0	120	68.6
AMC	23	13.1	0	0.0	152	86.9
FOX	123	70.3	0	0.0	52	29.7
KF	10	5.7	0	0.0	165	94.3
CTX	94	53.7	33	18.8	48	27.4
C	174	99.4	0	0.0	1	0.6
TE	39	22.3	58	33.1	78	44.6
SXT	172	98.3	0	0.0	3	1.7
ENR	124	70.9	27	15.4	24	13.7
CN	98	56.0	53	30.3	24	13.7
S	86	49.1	54	30.8	35	20.0
IPM	169	96.6	6	3.4	0	0.0
ATM	139	79.4	0	0.0	36	20.6

AM, ampicillin; AMC, amoxicillin–clavulanic acid; FOX, cefoxitin; KF, cephalothin; CTX, cefotaxime; C, chloramphenicol; TE, tetracycline; SXT, trimethoprim–sulfamethoxazole; ENR, enrofloxacin; CN, gentamicin; S, streptomycin; IPM, imipenem; ATM, aztreonam.

## Supplementary Materials

The following are available online at https://www.mdpi.com/2076-2615/10/4/744/s1: Table S1: Primers employed for detection of virulence genes; Table S2: Primers employed for detection of antimicrobial resistance genes; Table S3: Phenotypic and genotypic profiles of tested *E. coli* isolates.
