# Supplementary material for: Pathotypes and Antimicrobial Susceptibility of Escherichia Coli Isolated from Wild Boar (Sus scrofa) in Tuscany
Source: Animals (Basel). 2020 Apr 24;10(4):744. doi: 10.3390/ani10040744 (PMC7222796; doi:10.3390/ani10040744)
Supplement: Supplementary file 1 [file animals-10-00744-s001.pdf]

Supplementary Material

Table S1. Primers employed for detection of virulence genes.

Gene	Primer (5′-3′)	Product Size (bp)	Reference
escV	ATTCTGGCTCTCTTCTTCTTTATGGCTG<br>CGTCCCCTTTTACAAACTTCATCGC	544	Muller et al. (2007)
ent	TGGGCTAAAAGAAGACACACTG<br>CAAGCATCCTGATTATCTCACC	629	Muller et al. (2007)
eaeA	GACCCGGCACAAGCATAAGC<br>CCACCTGCAGCAACAAGAGG	384	Paton et al. (2002)
bfpB	GACACCTCATTGCTGAAGTCG<br>CCAGAACACCTCCGTTATGC	910	Muller et al. (2007)
stx1	ATAAATCGCCATTCGTTGACTAC<br>AGAACGCCCACTGAGATCATC	180	Paton et al. (2002)
stx2	GGCACTGTCTGAAACTGCTCC<br>TCGCCAGTTATCTGACATTCTG	255	Paton et al. (2002)
hlyA	GCATCATCAAGCGTACGTTCC<br>AATGAGCCAAGCTGGTTAAGCT	534	Paton et al. (2002)
saa	CGTGATGAACAGGCTATTGC<br>ATGGACATGCCTGTGGCAAC	119	Antikainen et al. (2009)
invE	CGATAGATGGCGAGAAATTATATCCCG<br>CGATCAAGAATCCCTAACAGAAGAATCAC	766	Muller et al. (2007)
astA	TGCCATCAACACAGTATATCCG<br>ACGGCTTTGTAGTCCTTCCAT	102	Muller et al. (2007)
aggR	ACGCAGAGTTGCCTGATAAAG<br>AATACAGAATCGTCAGCATCAGC	400	Muller et al. (2007)
pic	AGCCGTTTCCGCAGAAGCC<br>AAATGTCAGTGAACCGACGATTGG	1111	Muller et al. (2007)
elt	GAACAGGAGGTTTCTGCGTTAGGTG<br>CTTTCAATGGCTTTTTTTTGGGAGTC	655	Muller et al. (2007)
estIa	CCTCTTTTAGYCAGACARCTGAATCASTTG<br>CAGGCAGGATTACAACAAAGTTCACAG	157	Muller et al. (2007)
estIb	TGTCTTTTTCACCTTTCGCTC<br>CGGTACAAGCAGGATTACAACAC	171	Muller et al. (2007)

Table S1: Primers employed for detection of antimicrobial resistance genes.

Gene	Primer (5′-3′)	Product Size (bp)	Reference
bla$_{TEM}$	GTGGACAAAGGTACAACGAG<br>CGGTAAAGTTCGTCACACAC	857	(Maynard et al., 2003)
bla$_{CMY-2}$	GACAGCCTCTTTCTCCACA<br>TGGACACGAAGGCTACGTA	1000	(Maynard et al., 2003)
strA/*strB*	ATGGTGGACCCTAAAACTCT<br>CGTCTAGGATCGAGACAAAG	891	(Maynard et al., 2003)
aadA1	GTGGATGGCGGCCTGAAGCC<br>AATGCCCAGTCGGCAGCG	525	(Maynard et al., 2003)
tet(A)	GCTACATCCTGCTTGCCTTC<br>CATAGATCGCCGTGAAGAGG	210	(Dahshan, Shahada, Chuma, Moriki, & Okamoto, 2010)
tet(B)	TTGGTTAGGGGCAAGTTTTG<br>GTAATGGGCCAATAACACCG	659	(Dahshan et al., 2010)
tet(G)	GCTCGGTGGTATCTCTGCTC<br>AGCAACAGAATCGGGAACAC	468	(Dahshan et al., 2010)
sul1	TGGTGACGGTGTTCGGCATTC<br>GCGAGGGTTTCCGAGAAGGTG	789	(Costa et al., 2008)
sul2	CGGCATCGTCAACATAACC<br>GTGTGCGGATGAAAGTCAG	722	(Costa et al., 2008)
sul3	GAGCAAGATTTTTGGAATCG<br>CATCTGCAGCTAACCTAGGGTTTGGA	792	(Costa et al., 2008)

Table S2: Phenotypic and genotypic profiles of tested *E. coli* isolates

Isolate N°	Antimicrobial Resistance Profile	Antimicrobial Resistance Genes	Virulence Genes	Pathotype
C1	AM AMC KF	*sul2 strA/strB tet(B)*	*hlyA saa*	NC
C2	AM AMC KF TE	*sul2*		
C3	AMC KF		*stx1 stx2 hlyA*	EHEC
C4	AMC KF	*sul1 sul2*	*stx2 hlyA*	EHEC
C5	AM AMC KF	*tetA*	*stx2 hlyA astA aggR*	EHEC / EAEC
C6	KF			
C7	AMC	*bla_{CMY-2} sul1 tet(G)*	*stx2 hlyA astA*	EHEC
C8	AM AMC KF	*bla_{CMY-2} sul2 tet(G)*	*astA*	EAEC
C11	AM AMC KF		*pic*	EAEC
C12	AMC FOX KF CTX TE ATM	*sul2*	*hlyA*	NC
C14	AM AMC KF	*sul1 sul2 tet(B)*	*stx1 stx2 hlyA*	EHEC
C15	AMC KF ENR	*tetB*	*stx1 hlyA*	EHEC
C16	AM AMC FOX KF CTX TE ENR ATM	*sul2*	*stx2*	STEC
C17		*bla_{CMY-2} sul2 tet(G)*	*hlyA*	NC
C18	AM AMC KF	*sul1 sul2 sul3 tet(B)*		
C19	AM AMC KF	*sul2 tetG*		
C20	AM AMC FOX KF CTX ENR CN ATM	*sul1 tet(A) tet(G)*	*hlyA*	NC
C21	AM AMC FOX KF CTX ENR ATM	*sul2 sul3 tet(G)*	*eaeA hlyA astA*	EAEC/aEPEC
C22	AM AMC FOX KF CTX CN ATM	*sul2*	*eae stx1 hlyA*	EHEC
C23	AM KF	*sul2 tetG*		
C24	AM AMC FOX KF CTX TE CN ATM	*bla_{CMY-2} sul1 tet(B) tet(G)*		
C25	AM AMC FOX KF CTX TE ENR ATM	*bla_{CMY-2} sul1 tet(B)*		
C26	AMC FOX KF CTX TE ATM	*sul1*		
C27	AM AMC FOX KF CTX TE CN ATM	*bla_{CMY-2} sul1 tet(G)*		
C28	AM AMC FOX KF CTX TE ENR CN ATM	*bla_{CMY-2} sul1 sul2 tet(G)*		
C29	AM AMC KF	*bla_{CMY-2} tet(G)*	*stx1 astA*	STEC

C30	AM AMC FOX KF CTX TE CN ATM	*bla*$_{CMY-2}$ *sul1 sul2 tet(G)*	*hlyA astA*	NC
C31	AM AMC KF	*bla*$_{CMY-2}$ *tet(G)*	*hlyA astA*	NC
C32	AM AMC KF	*bla*$_{CMY-2}$ *tet(G)*	*hlyA astA*	NC
C33	AMC KF	*tet(A) tet(G)*	*eaeA hlyA astA*	EAEC/aEPEC
C34	AM AMC FOX KF CTX TE ENR ATM	*tet(B)*	*astA*	EAEC
C35	AM FOX KF CTX ENR CN ATM	*sul2*		
C36	AM AMC FOX KF CTX			
C37		*bla*$_{CMY-2}$ *sul2 tet(G)*	*hlyA*	NC
C38	AM AMC KF TE	*sul2 strA/strB tet(G)*	*eaeA astA*	EPEC/EAEC
C39	AMC KF	*sul2 sul3 tet(B)*	*hlyA astA*	NC
C41	AM AMC FOX KF CTX CN ATM	*sul2*		
C43	AM AMC FOX KF CTX TE ENR CN ATM	*sul1 sul2 tet(G)*	*hlyA*	NC
C45	AM AMC KF	*tetG*		
C46	AM KF	*sul2 tetG*		
C47	AM AMC KF TE			
C48	AMC KF CTX CN ATM	*strA/strB tetA tetG*		
C49	AM AMC FOX KF CTX TE CN ATM			
C50	AM AMC FOX KF ATM	*sul1 sul2*		
C51	AM AMC KF TE	*sul3*	*eaeA stx2 hlyA*	EHEC
C52	AM AMC FOX KF TE ENR	*sul2*	*hlyA astA*	NC
C53	AM AMC KF			
C54	AMC FOX KF CTX ENR CN ATM			
C55	AM AMC FOX KF TE	*tetB*	*hlyA astA*	NC
C56	AM AMC KF TE	*sul1*		
C57	AM AMC FOX KF CTX TE ENR CN ATM		*stx1 hlyA*	EHEC
C58	AMC KF TE	*tetA*		
C59	AM AMC KF CTX TE	*bla*$_{CMY-2}$ *sul1 sul2 tet(G)*	*eaeA hlyA escV astA aggR*	EAEC/aEPEC
C60	FOX KF CTX TE ATM		*pic*	EAEC
C61	AMC FOX KF CTX TE CN ATM	*sul2 tet(B)*	*eaeA pic*	EPEC/EAEC

C62	AM AMC FOX KF CTX CN ATM	*tetB*	*hlyA astA*	NC
C63	AM AMC FOX KF CTX CN ATM	*tetB*		
C64	AM AMC KF	*tetA*		
C65	AM AMC KF	*sul2 tetG*		
C67	AM AMC KF ENR	*sul2*		
C70	AM AMC KF ENR			
C71	AM AMC FOX KF CTX TE SXT ENR CN ATM	*sul1 sul2*		
C72	AM AMC KF	*bla_{CMY-2} sul1*	*stx2 hlyA*	EHEC
C73	AM FOX KF CTX CN ATM	*bla_{CMY-2} tet(G)*	*stx2 astA*	STEC
C74	AM FOX KF CTX TE ATM	*bla_{CMY-2}*		
C75	AM KF ENR	*sul1 sul2 tet(G)*	*stx2*	STEC
C76	AM AMC FOX KF	*bla_{CMY-2}*	*stx2*	STEC
C77	KF CTX ATM	*sul2 tet(B)*	*stx2 hlyA*	EHEC
C78	AM AMC KF			
C79	AM AMC FOX KF CTX	*sul2*	*hlyA*	NC
C81	AM AMC FOX KF	*sul2*		
C82	TE	*sul1*		
C83	AM AMC FOX KF CTX TE	*tetB*	*eaeA*	aEPEC
C84	AM AMC FOX KF TE		*hlyA saa*	NC
C85	AM AMC KF TE			
C87	AM AMC KF TE	*sul1 sul2strA/strB tet(G)*		
C89	AM AMC KF	*sul2*		
C90	AM AMC KF CTX TE SXT ENR ATM	*strA/strB tetA tetG*		
C91	AM AMC FOX KF		*stx2 astA*	STEC
C92	AM AMC FOX KF TE	*bla_{CMY-2} sul2 aadA1*	*eaeA*	aEPEC
C93	AMC TE	*bla_{CMY-2} sul1 sul2*	*astA*	EAEC
C94	AMC FOX KF	*bla_{CMY-2} sul1 sul2 tet(G)*	*eaeA escV astA aggR*	EHEC/EAEC
C95	AM AMC KF TE		*stx2 astA*	STEC
C96	AMC	*bla_{CMY-2} aadA1 tet(G)*	*hlyA*	NC
C97	AMC KF	*sul1 sul2 aadA1 tet(B)*	*stx1 stx2*	STEC
C98	KF	*bla_{CMY-2} sul1 sul2 aadA1 tet(G)*	*stx2 astA aggR*	STEC/EAEC
C99	AM AMC KF ENR	*bla_{CMY-2} sul1 sul2*	*hlyA astA*	NC
C100	AMC KF	*bla_{CMY-2} sul1 tet(G)*	*hlyA*	NC

C101	AMC FOX KF	*bla*$_{CMY-2}$ *tet(A)*	*escV*	aEPEC
C103	AM AMC KF	*sul1 aadA1 tet(A) tet(G)*	*stx2 hlyA astA*	EHEC
C104	AM AMC FOX KF CTX TE	*bla*$_{CMY-2}$ *sul1 sul2*	*hlyA*	NC
C105	AM AMC KF CTX TE	*bla*$_{CMY-2}$ *sul1 strA/strB tet(A) tet(B) tet(G)*	*eaeA hlyA*	aEPEC
C106	AM AMC KF TE	*bla*$_{CMY-2}$ *sul1 tet(A) tet(B) tet(G)*	*hlyA astA*	NC
C108	AM AMC KF CTX TE ENR	*tetA tetG*	*astA*	EAEC
C109	AM AMC KF TE	*bla*$_{CMY-2}$ *sul1 sul2 aadA1*	*hlyA*	NC
C110	AM AMC KF TE	*bla*$_{CMY-2}$*sul1 tet(A) tet(G)*	*stx1 hlyA*	EHEC
C112	AM AMC KF	*bla*$_{CMY-2}$ *sul2 tet(B)*	*hlyA*	NC
C113	AMC KF	*bla*$_{CMY-2}$ *sul1 aadA1 tet(G)*	*stx2*	STEC
C115	AM KF	*bla*$_{CMY-2}$ *tet(G)*	*eaeA hlyA escV*	aEPEC
C116	KF	*bla*$_{CMY-2}$ *tet(A)*	*eaeA hlyA astA*	EAEC/aEPEC
C117	AM AMC KF TE	*bla*$_{CMY-2}$ *sul1 tet(A) tet(B)*	*hlyA astA*	NC
C118	AM AMC KF	*tetA tetB tetG*	*stx2 astA*	STEC
C119	AMC KF CTX	*bla*$_{CMY-2}$ *sul1 tet(A)*		
C120	AMC KF	*bla*$_{CMY-2}$ *tet(A)*		
C122	AMC KF	*bla*$_{CMY-2}$ *strA/strB tet(A)*		
C123	AM AMC KF	*bla*$_{CMY-2}$ *sul1 aadA1 tet(A) tet(G)*	*stx2*	STEC
C124	AM AMC KF	*bla*$_{CMY-2}$	*eaeA stx2 hlyA escV astA aggR*	EHEC/EAEC /aEPEC
C125	AM AMC KF TE	*bla*$_{CMY-2}$ *sul1 tet(A)*	*hlyA escV astA aggR*	EAEC/aEPEC
C126	AM AMC KF TE	*bla*$_{CMY-2}$ *aadA1*	*stx2 hlyA astA*	EHEC
C127	AM KF TE	*bla*$_{CMY-2}$ *sul2*	*hlyA*	NC
C128	AM AMC KF TE	*bla*$_{CMY-2}$ *sul1 sul2*	*stx2 astA*	STEC
C129	AM AMC KF CTX TE	*bla*$_{CMY-2}$ *tet(A)*	*eaeA*	aEPEC
C130	AM AMC FOX KF CTX TE CN ATM	*bla*$_{CMY-2}$ *sul1*	*stx2 astA*	STEC
C131	AM AMC FOX KF CTX TE	*bla*$_{CMY-2}$ *tet(A) tet(G)*		
C132	AMC FOX KF CTX CN ATM	*tetA tetG*		
C133	AM AMC FOX KF CTX CN ATM	*bla*$_{CMY-2}$		
C134	AM AMC KF TE	*bla*$_{CMY-2}$ *sul1*	*hlyA*	NC
C135	AM AMC KF TE	*bla*$_{CMY-2}$ *sul1 strA/strB tet(G)*	*hlyA*	NC
C136	AM AMC KF	*bla*$_{CMY-2}$		

C138	AM AMC KF ENR			
C139	AM AMC KF	*bla*$_{CMY-2}$ *sul1 aadA1*	*stx2 hlyA pic*	EHEC/EAEC
C140	AM AMC KF TE	*bla*$_{CMY-2}$ *sul1 tet(A)*	*stx2 hlyA astA*	EHEC
C141	AM AMC KF TE	*bla*$_{CMY-2}$ *sul2 strA/strB*	*stx2 hlyA*	EHEC
C142	AM AMC FOX KF CTX TE ENR CN ATM	*bla*$_{CMY-2}$ *sul1 tet(A)*	*stx2 hlyA*	EHEC
C143	AM AMC FOX KF TE	*bla*$_{CMY-2}$ *sul1*	*pic*	EAEC
C144	AM AMC KF TE	*bla*$_{CMY-2}$ *sul1 sul2*	*stx2*	STEC
C145	AM AMC KF CTX TE ENR		*stx2 astA*	STEC
C146	AM AMC FOX KF CTX TE ENR CN ATM	*bla*$_{CMY-2}$		
C147	AM AMC FOX KF CTX TE ENR CN ATM	*bla*$_{CMY-2}$ *strA/strB tet(B)*	*eaeA escV*	aEPEC
C148	AMC KF CTX	*bla*$_{CMY-2}$ *sul1*	*stx2 hlyA*	EHEC
C149	AM AMC KF TE			
C150	AM KF TE	*bla*$_{CMY-2}$ *sul1 tet(B)*	*hlyA astA*	NC
C151	AM AMC KF TE	*bla*$_{CMY-2}$ *sul1*		
C152	AM AMC KF		*hlyA astA*	NC
C153	AM AMC KF TE	*bla*$_{CMY-2}$ *sul1*	*hlyA astA*	NC
C154	AM AMC KF CTX TE	*bla*$_{CMY-2}$ *sul1 tet(B)*	*hlyA pic (AIEC)*	NC
C155	AM AMC KF TE	*bla*$_{CMY-2}$ *sul1 aadA1*	*hlyA*	NC
C156	AM AMC KF TE	*bla*$_{CMY-2}$ *sul1*	*eaeA hlyA astA pic*	EAEC/aEPEC
C159	AM AMC KF TE	*bla*$_{CMY-2}$ *sul1*		
C160	AMC KF	*bla*$_{CMY-2}$ *sul1 aadA1*	*eaeA hlyA astA*	EAEC/aEPEC
C161	AMC KF	*bla*$_{CMY-2}$ *sul1*	*eaeA stx2 hlyA*	EHEC
C163	AMC FOX KF	*bla*$_{CMY-2}$ *sul1*	*hlyA pic (AIEC)*	NC
C164	FOX	*bla*$_{CMY-2}$ *sul1 sul2 tet(A)*	*hlyA*	NC
C165	AMC KF	*bla*$_{CMY-2}$ *sul1 sul2 tet(A)*	*hlyA astA*	NC
C167	AM AMC KF	*bla*$_{CMY-2}$	*stx2 hlyA astA*	EHEC
C168	AM AMC KF TE	*bla*$_{CMY-2}$ *sul2*		
C169	AM AMC KF	*bla*$_{CMY-2}$		
C171	AM AMC KF CTX	*sul1 sul2*	*pic*	EAEC
C172	AMC KF TE	*bla*$_{CMY-2}$ *sul1 tet(B)*		
C173	AM AMC FOX KF	*bla*$_{CMY-2}$ *sul1 tet(A)*	*stx2 hlyA*	EHEC
C174	AMC KF TE	*bla*$_{CMY-2}$ *sul1*	*stx2*	STEC
C175	AMC KF TE	*bla*$_{CMY-2}$ *sul1 tet(B)*	*stx2*	STEC
C176	AM AMC KF TE	*bla*$_{CMY-2}$ *sul1 aadA1*	*stx2 hlyA*	EHEC
C177	AMC KF TE	*bla*$_{CMY-2}$ *sul1*	*stx2 hlyA*	EHEC

C178	AM AMC KF TE	*bla*$_{CMY-2}$ *sul1 aadA1 tet(A)*	*hlyA*	NC
C179	AM AMC KF TE	*bla*$_{CMY-2}$ *aadA1*	*eae stx1 stx2 hlyA*	EHEC
C180	AMC KF	*bla*$_{CMY-2}$	*eae stx1 hlyA*	EHEC
C181	AMC FOX KF TE	*bla*$_{CMY-2}$ *aadA2*	*eae stx1 hlyA*	EHEC
C182	AMC	*bla*$_{CMY-2}$ *sul2*	*eae stx1 hlyA*	EHEC
C183	AMC KF	*bla*$_{CMY-2}$ *sul1 aadA1*	*eae stx1 hlyA astA pic*	EHEC/EAEC
C184	AMC KF	*bla*$_{CMY-2}$	*eae stx1 hlyA astA pic*	EHEC/EAEC
C185	AMC	*bla*$_{CMY-2}$	*eae stx1 hlyA*	EHEC
C186	AM AMC KF	*bla*$_{CMY-2}$ *sul2*	*eae stx1 hlyA*	EHEC
C187	AM AMC KF	*bla*$_{CMY-2}$ *aadA1 tet(A)*	*stx1 stx2 hlyA*	EHEC
C189	AMC KF	*bla*$_{CMY-2}$ *tet(A)*	*astA*	EAEC
C190	KF TE			
C191	AMC KF		*eaeA hlyA*	aEPEC
C192	KF	*bla*$_{CMY-2}$ *aadA1*	*hlyA astA*	NC
C193	KF		*hlyA astA*	NC
C194	AM AMC FOX KF C TE SXT		*eae stx1 stx2*	EHEC
C195	AMC KF	*bla*$_{CMY-2}$		
C197	AM AMC KF		*astA*	EAEC
C198	KF ATM	*bla*$_{CMY-2}$	*eae stx1 stx2 hlyA escV*	EHEC
C199	AM AMC KF TE		*stx1 stx3*	STEC
C200			*hlyA astA*	NC

Legend: AM = ampicillin; AMC = amoxicillin/clavulanic acid; FOX = cefoxitin; KF = cephalothin; CTX = cefotaxime; C = chloramphenicol; TE = tetracycline; SXT = trimethoprim/sulfamethoxazole; ENR = enrofloxacin; CN = gentamicin; S = streptomycin; IPM = imipenem; ATM = aztreonam; NC = Not classifiable
